# Study on Properties and Micro-Mechanism of RHB-SBS Composite-Modified Asphalt

**DOI:** 10.3390/polym15071718

**Published:** 2023-03-30

**Authors:** Youqiu Yi, Yifan Chen, Shuo Shi, Yao Zhao, Daming Wang, Tao Lei, Pengpeng Duan, Weiwei Cao, Qiang Wang, Haitao Li

**Affiliations:** 1 College of Civil Engineering, Nanjing Forestry University, Nanjing 210037, China; 2 Nanjing Freetech Road Recycling Co., Ltd., Nanjing 320100, China

**Keywords:** agricultural waste, rice husk biochar, SBS, composite-modified asphalt, rheological behaviors, microscopic features

## Abstract

Rice husk biochar (RHB) is a renewable agricultural waste, and its fixation on pavements helps develop environmentally friendly, economical, and sustainable asphalt pavements. This paper used RHB to replace part of styrene-butadiene-styrene (SBS) for the composite modification study of matrix asphalt. The high- and low-temperature properties and microscopic mechanisms of the composite-modified asphalt were studied through a series of tests. The results showed that, compared with SBS-modified asphalt, the softening point, viscosity, complex shear modulus, stiffness modulus, and rutting factors of RHB-SBS composite-modified asphalt were improved. In contrast, the ductility and creep rate were slightly decreased, indicating an improvement in the high-temperature performance of composite-modified asphalt, but a slight decrease in its low-temperature performance. The process of RHB and SBS composite modification was mainly physical blending, with only a small number of chemical reactions, and no new functional groups were generated. The porous structure of RHB enables it to adhere better to the network crosslinked continuous phase system formed by SBS and matrix asphalt. This results in composite-modified asphalt with good high-temperature storage stability and rheological properties. Therefore, RHB-SBS composite-modified asphalt can be applied to high-temperature areas and rice-producing areas, and the optimal content of RHB is suggested to be 15%.

## 1. Introduction

To alleviate the pressure of non-renewable energy shortage and contribute to the “double carbon” goal, an economical, practical, and environmentally friendly composite material is needed to modify asphalt [1]. Compared with matrix asphalt, SBS-modified asphalt shows great advantages in various properties under high-temperature and low-temperature conditions. At the present, SBS-modified asphalt is still the mainstream option when paving high-grade highway pavements at home and abroad. However, the defects of SBS-modified asphalt, such as the high initial cost, complex processing, poor storage stability, and high-temperature performance, cannot meet the requirements of some areas, limiting its further development to a certain extent. In terms of stability, Dong et al. [2] modified rubber powder and SBS composite, finding that the resulting rubber powder and SBS composite-modified asphalt had a good storage stability. Zhang et al. [3] found that adding 0.2% sulfur to SBS composite-modified asphalt could further improve its storage stability and flexibility. The content of SBS substituted by rubber powder is limited, and it is unclear whether toxic sulfide is generated when sulfur is added to SBS composite-modified asphalt. To reduce the content of SBS in asphalt, Song [4] et al. studied the feasibility of replacing part of SBS with crumb rubber (CR) to improve the high- and low-temperature performance of asphalt. The results showed that the higher the temperature and longer the time during the preparation of CR and SBS composite-modified asphalt, the better the high-temperature performance, but the increase in temperature and time would be detrimental to the low-temperature performance. Many researchers have attempted to compositely modify matrix asphalt with SBS modifiers and various agents [5,6], such as poly phosphoric acid (PPA), reactive ethylene terpolymer (RET), and high modulus agents, to enhance its overall performance and economic benefits. However, the results were not satisfactory.

Due to the imbalance between the supply and demand of fossil energy and the need to reduce emissions and the cost of materials, researchers are beginning to focus on large amounts of waste renewable biomass resources. Biomass raw materials can be converted into gas (pyrolysis gas), liquid (bio-oil), and solid products (biochar) with high energy density by low-cost and efficient pyrolysis technology [7]. Common biochar includes charcoal, bamboo charcoal, rice husk charcoal, straw charcoal, and so on [8]. However, rice husk waste, caused by processing rice in our country every year, is abundant and low cost [9]. Rice husk biochar (RHB) with a rich carbon content is obtained by pyrolysis under temperatures less than 700 °C and without oxygen [10]. Compared with biochar from other sources, rice husk biochar has a smaller average particle size (5–10 μm), higher specific surface activity (50–60 m^2^/g), and a more extensive amorphous silica content (80–95%) [11]. It has the characteristics of hardness, good wear resistance, light-weight, low thermal conductivity, high content of active silicon, large specific surface area, and porosity [12].

At the present, there are few studies on RHB and SBS composite-modified asphalt. However, many scholars have prepared modified asphalt by adding rice husk ash (RHA), which has the same raw material and similar composition structure as rice husk biochar, into matrix asphalt. Xue et al. [13] found no chemical reaction between RHA and matrix asphalt, and the primary modification mode was physical co-blending. After observing the microstructure of RHA, Han et al. [14] found that RHA had a porous structure with a rough surface, and the interlayer was composed of staggered wafers and pores. This structure is conducive to the absorption of matrix asphalt. The three-dimensional mesh micro-wafers on the surface of RHA particles and the white columnar silica crystals on the edges also have an attractive effect on the matrix asphalt. RHA and matrix asphalt can form a stable three-dimensional network system, which is conducive to improving the high-temperature deformation resistance and bonding properties of asphalt. Lu et al. [15] selected different contents of RHA and 1% SBS to prepare modified asphalt and conducted a series of macro tests on asphalt and asphalt mixture. The results showed that the high-temperature performance of the RHA and SBS composite-modified asphalt mixture was better, and the water stability and low-temperature performance were slightly lower, compared with the matrix asphalt. However, Lu et al. only used a single dose of SBS and did not analyze the modification mechanism of RHA and SBS composite-modified asphalt from a microscopic perspective. Numerous studies have shown the beneficial effect of RHA on the performance of asphalt. However, RHA is the waste product of rice husk burning under aerobic conditions, which produces large amounts of carbon dioxide and other gases that are harmful to the environment. Therefore, in this paper, based on the research of biochar and RHA, RHB was incorporated into the asphalt as a new type of modifier for agricultural waste treatment. Furthermore, RHB, a solid carbonaceous residue, can be fixed on the asphalt pavement.

In this study, RHB prepared from agricultural waste rice husk was used to replace part of SBS to study the temperature sensitivity, rheological properties, high- and low-temperature properties, and micro-mechanisms of RHB and SBS composite-modified asphalt. Firstly, the asphalts were evaluated by measuring the penetration, softening point, ductility, and viscosity, and the rheological behavior of asphalt was evaluated by temperature scanning test with a dynamic shear rheometer. Secondly, the low-temperature performance of the asphalt was analyzed using a bending beam rheometer test and the verification analysis of road performance was carried out. Finally, Fourier transform infrared spectroscopy, environmental scanning electron microscopy, and Leica stereo fluorescence microscopy were used to analyze the properties and micro-mechanisms of RHB, SBS, and their composite-modified asphalt.

## 2. Materials and Methods

### 2.1. Asphalt

In this study, 70# matrix asphalt was produced by Alpha Asphalt Co., LTD., (Jiangyin, Nanjing, China), The test results of penetration, softening point, ductility, and dynamic viscosity are presented in Table 1 according to the Test Regulations for Highway Engineering Asphalt and Asphalt Mixture (JTG E20-2011) [16].

### 2.2. SBS

**Figure 1:**
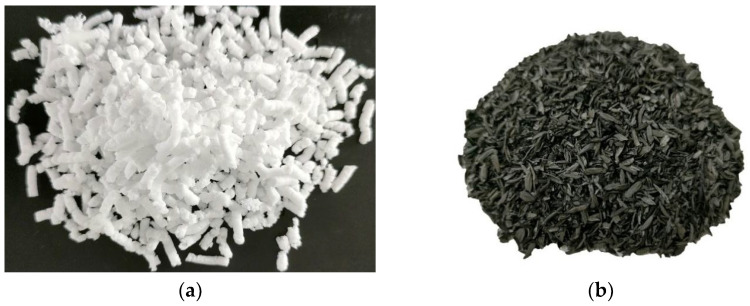
(**a**) Apparent morphology of SBS, (**b**) apparent morphology of rice husk biochar.

The adopted SBS was Baling star structure YH-791-H, with a block ratio of 3:7, and its appearance was white stripe particles, as shown in Figure 1a. The basic physical properties are shown in Table 2.

### 2.3. RHB

The RHB used in the study was produced by Jiangxi Shengniu Rice Industry Co., LTD. (Shangrao, China), using rice husk as the raw material and undergoing gasification and co-production of carbon. It has a black appearance, as shown in Figure 1b. The contents of moisture, ash, volatiles, and fixed carbon were determined according to the requirements, and the elemental analyzer was used to analyze RHB. The test results are shown in Table 3. The proportion of carbon in RHB is more than half, and the proportion of oxygen is also significant. Its composition contains a substantial amount of ash and fixed carbon. Other basic technical indicators of RHB are displayed in Table 4.

### 2.4. Sample Preparation Method

The content of RHB was 7.5%, 10%, 12.5%, 15%, 17.5%, and 20% of the total weight of the matrix asphalt, and the content of SBS was 2.0%, 2.5%, 3.0%, 3.5%, 4%, and 4.5% of the total weight of the matrix asphalt. The two modifiers were orthogonally tested, and a total of 36 matching samples were obtained. After conventional testing, comprehensive comparison, and a statistical analysis of composite-modified asphalt, the mass fraction of RHB was 15%, and the content of SBS was 0%, 2.0%, 2.5%, 3.0%, 3.5%, 4%, and 4.5%, respectively. The mass fraction of SBS was 4% and the content of RHB was 0%, 7.5%, 10%, 12.5%, 15%, 17.5%, and 20%, respectively. A total of 14 proportion combinations were selected to study the influence of RHB and SBS on the rheological characteristics and microstructure changes of matrix asphalt.

**Figure 2:**
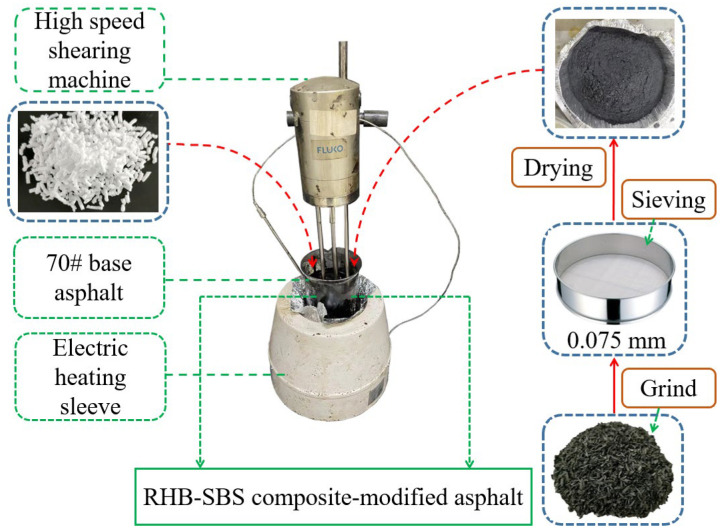
Flow chart of preparation of composite-modified asphalt.

The samples preparation process is shown in Figure 2.

### 2.5. Experimental Method

#### 2.5.1. Conventional Physical Performance Experiments

The penetration test of asphalt binder was carried out at 15 °C, 25 °C, and 35 °C, which was performed in accordance with JTG E20-2011 T0604. The softening point of asphalt was determined according to the JTG E20-2011 T0606. For the ductility test, 10 °C was used as the test temperature, 5 cm/min was used as the tensile speed, and the ductility test was carried out according to JTG E20-2011 T0605. According to JTG E20-2011 T0620, the dynamic viscosity of asphalt at 60 °C was determined by the vacuum capillary method.

#### 2.5.2. Rheological Property Experiments

The dynamic shear rheometer (DSR) can be used to conduct temperature scanning tests on asphalt samples to study dynamic viscoelastic parameters such as complex shear modulus (G*/kPa), phase angle (δ/°), and rutting factor (G*/sinδ) at different temperatures and the same frequency. This can further help evaluate the temperature sensitivity of asphalt. The test was conducted according to the AASHTO T315 [17] standard, with a frequency of 10 rad/s and an accuracy of 1%. The diameter of the test plate was (25.00 ± 0.05) mm and the clearance was 1 mm. The temperature scanning range was 46 °C to 82 °C, with a temperature interval of 6 °C. The bending stiffness modulus and m-value of the asphalt samples were tested by the bending beam rheometer (BBR) at −12 °C to study the low-temperature crack resistance of the asphalt binder. This was performed in accordance with AASHTO T313 [18].

#### 2.5.3. Microscopic Experiments

In this paper, a VERTEX 80V Fourier transform infrared spectroscopy (FTIR) from Bruker, Germany was used to scan the asphalt modifiers and modified asphalt. After absorbing infrared radiation, dipole moment changes and vibrational energy level transitions occur in molecules, resulting in infrared spectral absorption bands [19]. Most compounds possess a unique infrared absorption spectrum. The structure information of known compounds can be analyzed, and unknown compounds can be identified by the observation of infrared spectrum [20]. The KBR pellet pressing method was used to test RHB: a certain amount of rice husk charcoal powder and potassium bromide powder was mixed and ground to the micron level, pressed into a translucent sheet, and then analyzed using an infrared spectrometer. SBS and composite-modified asphalt were tested using the ATR annex method: SBS was placed on a glass slide (the asphalt was heated and dropped onto the glass slide), cooled, and analyzed using an infrared spectrometer.

The microscopic morphology of the modifier and the composite-modified asphalt were observed using an environmental scanning electron microscope (SEM), Quanta 200, FEI, Hillsboro, OR, USA. The electron beam in the SEM interacts with the sample as it scans the sample surface, which excites various physical signals [21]. The instrument detects, processes, and amplifies these signals to obtain a scanned image of the sample microform [22]. The composite-modified asphalt was placed on a sample carrier and sputtered with gold spray under vacuum conditions. The microscopic morphology was observed using a scanning electron microscope.

The Leica M205FA stereo fluorescence microscope produced by the Leica company in Germany was used to observe the distribution of the polymer phase in composite-modified asphalt, which is beneficial for analyzing the compatibility of the internal system of composite-modified asphalt. The fluorescence microscope was used to observe the shape and position of the fluorescence reflected from the sample under the microscope after irradiating the sample with ultraviolet light. The excitation wavelength used in this test was (48 ± 20) nm and the emission wavelength was 510 nm.

## 3. Results and Discussion

### 3.1. Conventional Properties of RHB-SBS Composite-Modified Asphalt

#### 3.1.1. Penetration Experiment

**Figure 3:**
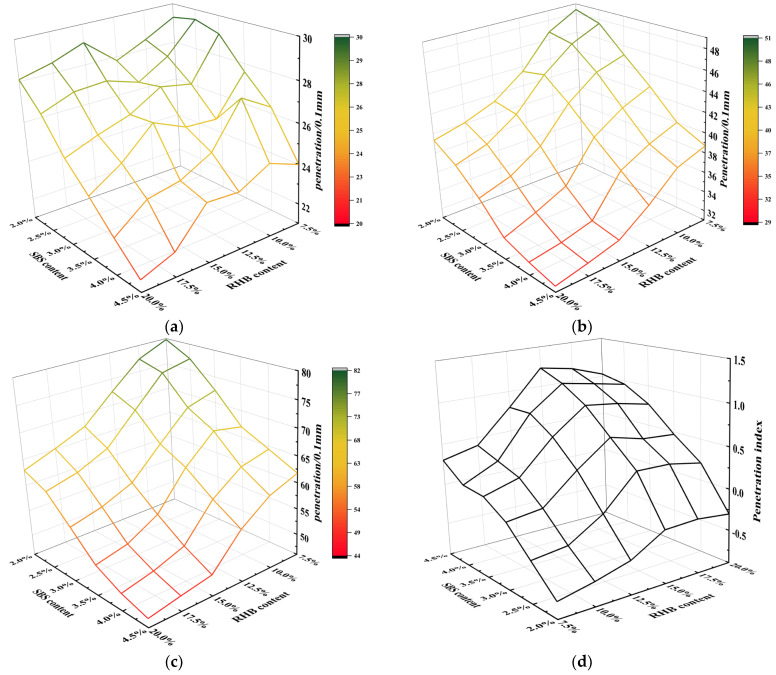
Penetration and penetration index of composite-modified asphalt at different temperatures. (**a**) 15 °C penetration, (**b**) 25 °C penetration, (**c**) 30 °C penetration, (**d**) penetration index.

In this paper, the penetration of RHB-SBS composite-modified asphalt was tested under three different temperature conditions of 15 °C, 25 °C, and 30 °C, and the results are shown in Figure 3a–c.

As shown in Figure 3a–c, with the increase in RHB and SBS content, the penetration of asphalt presents a downward trend on the whole. The penetration decreases significantly when the RHB content is 10~15% and the SBS content is 2.5~4%.

To further study the effect of RHB and SBS on the temperature sensitivity of asphalt, the penetration index was calculated according to the formula in the test procedure JTG E20-2011 T0604, and the calculation results are shown in Figure 3d. As the content of SBS in the composite-modified asphalt increased, the penetration index increased continuously. When the content of RHB increased, the penetration index increased first and then began to decline slowly when the content of RHB exceeded 15%. The results indicated that the increase in SBS proportion in composite-modified asphalt can significantly reduce the influence of temperature change on the performance of asphalt, thereby decreasing its temperature sensitivity and improving its temperature stability.

#### 3.1.2. Softening Point Experiment

**Figure 4:**
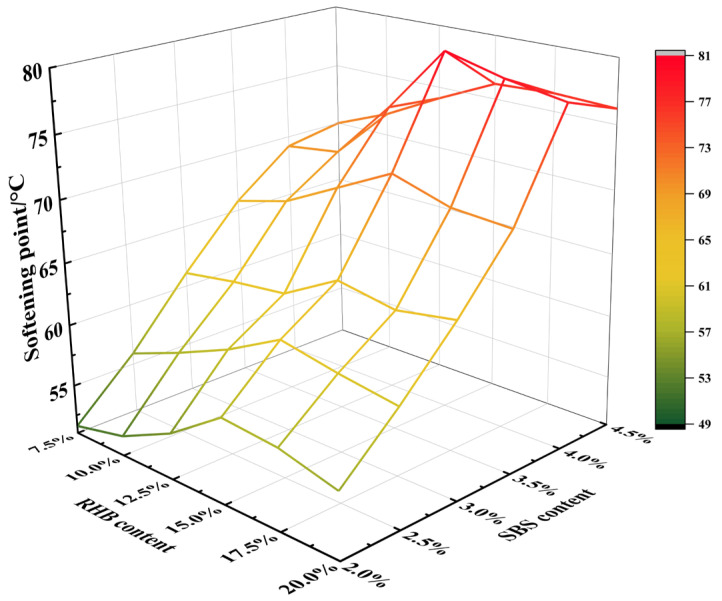
Softening point of composite-modified asphalt.

As seen in Figure 4, the increase in the content of RHB and SBS makes the softening point of asphalt continuously improve on the whole, and the high softening point indicates that asphalt has a better flow resistance at high temperatures. Among them, when the content of RHB is 15% and SBS is 4%, the softening point of the composite-modified asphalt reaches its maximum value. Thereafter, when the content of RHB or SBS is increased, the softening point only slightly increases or decreases, indicating that there is an optimal content of RHB and SBS in the asphalt only for the softening point of composite-modified asphalt. When the optimal amount is reached, the composite-modified asphalt has the best high-temperature resistance and high-temperature performance. The optimal amount is when the content of RHB is 15%, and the content of SBS is 4%.

#### 3.1.3. Ductility Experiment

**Figure 5:**
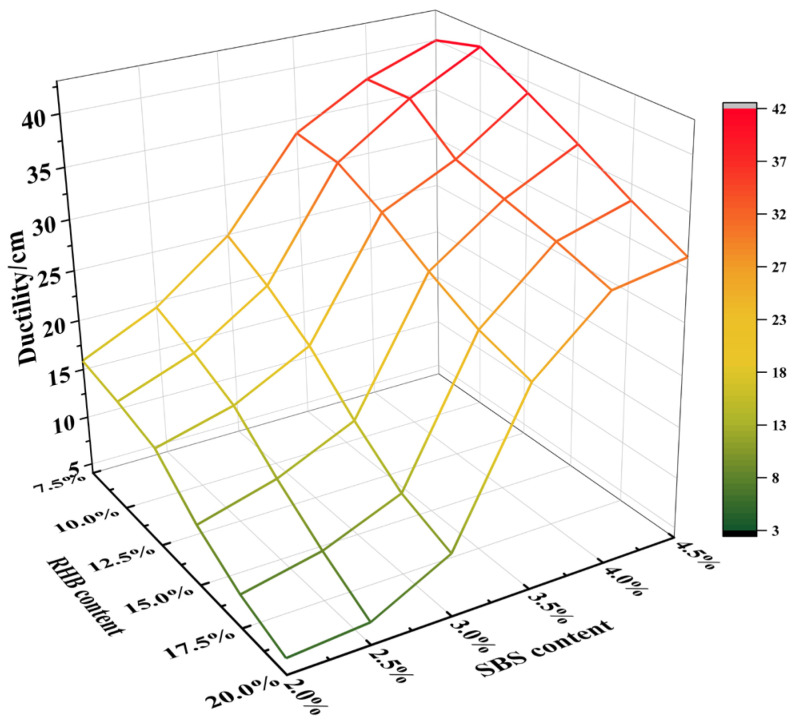
Ductility of composite-modified asphalt.

The ductility experiment of composite-modified asphalt samples with different dosages were carried out at the temperature of 10 °C, and the results are shown in Figure 5.

Figure 5 shows that the overall trend of the ductility of the composite-modified asphalt is reduced. Furthermore, in the case of the constant amount of SBS, an increase in the content of RHB leads to a decrease in the ductility of the asphalt. Conversely, in the case of a constant amount of RHB, an increase in SBS content increases the ductility of the asphalt. The results indicate that the incorporation of RHB has a negative impact on the low-temperature properties of the composite-modified asphalt. However, the incorporation of SBS can mitigate this negative impact.

#### 3.1.4. Dynamic Viscosity Experiment

**Figure 6:**
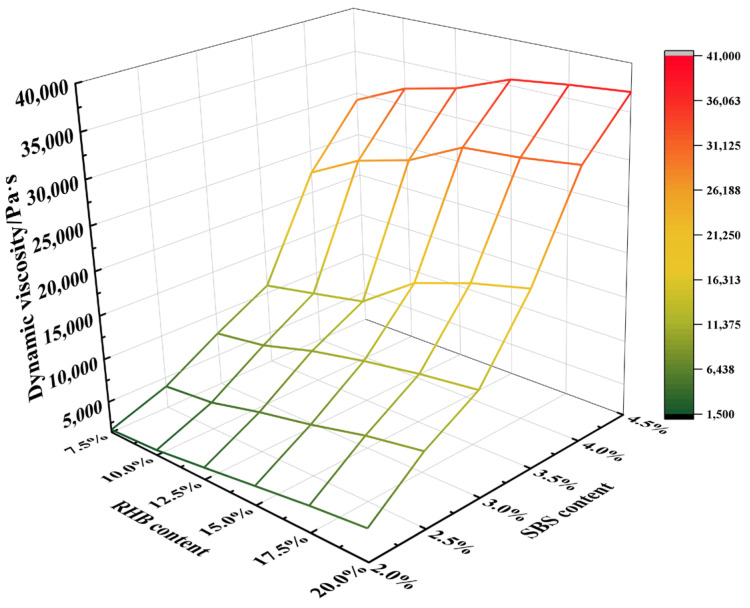
Dynamic viscosity of composite-modified asphalt.

The dynamic viscosity of the composite-modified asphalt was tested using a dynamic viscosity tester at 60 °C, and the test results are shown in Figure 6.

As seen from Figure 6, with the increase in the dosage of RHB and SBS modifier, the dynamic viscosity of the composite-modified asphalt improves constantly. Its value is far more than the 180 Pa·s required by the specification, indicating that the RHB-SBS composite-modified asphalt has a good stability and durability.

### 3.2. Rheological Properties of RHB-SBS Composite-Modified Asphalt

#### 3.2.1. Temperature Sweep Experiment

**Figure 7:**
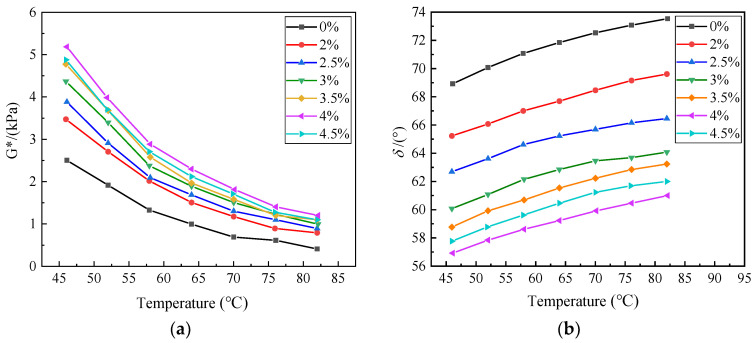
(**a**) The complex shear modulus of different content of SBS, (**b**) phase angle of different contents of SBS.

The complex shear modulus (G*) is a measure of the total resistance of a material to repeated shear deformation. A higher complex modulus indicates a stronger anti-deformation ability of asphalt. The phase angle indicates the viscoelastic ratio of asphalt at high temperatures. A larger phase angle indicates higher viscosity of the asphalt and weaker elastic recovery of high-temperature deformation [23]. The results are shown in Figure 7a,b.

The change of the complex shear modulus (G*) of RHB-SBS composite-modified asphalt with temperature at different SBS doping levels is shown in Figure 7a, which shows that the complex shear modulus (G*) decreases gradually with the increase in temperature, and the higher the temperature, the smaller the difference of complex shear modulus (G*) between the doping levels. The complex shear modulus (G*) tends to increase with the increase in the SBS content in the range of 0% to 4% and decreases when the SBS content increases to 4.5%. In Figure 7b, the phase angle of RHB-SBS composite-modified asphalt varies with temperature under the same RHB and different SBS content. With the increase in the temperature, the phase angle gradually increases, and with the increase in the SBS content, the phase angle becomes smaller and smaller. When the SBS content is 4.5%, the phase angle slightly increases. A smaller phase angle indicates lower high-temperature viscosity of asphalt, which is more conducive to elastic recovery. Overall, when the content of RHB is constant and the content of SBS is 4%, the complex shear modulus (G*) is the largest, the phase angle is the smallest, and the obtained composite-modified asphalt has the best resistance to high-temperature deformation.

**Figure 8:**
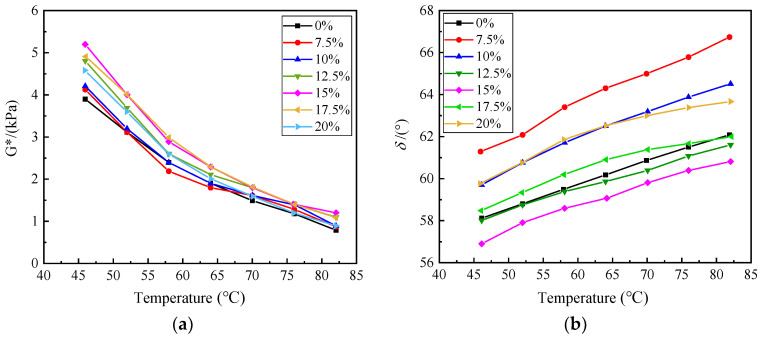
(**a**) The complex modulus of different content of RHB, (**b**) phase angle of different content of RHB.

The curves of the complex shear modulus (G*) and the phase angle versus the temperature for the same content of SBS and different content of RHB are shown in Figure 8a,b, respectively. The figures indicate that the complex shear modulus (G*) gradually decreases and the phase angle gradually increases with an increase in temperature. When the content of SBS is fixed and the content of RHB increases from 7.5% to 15%, the complex shear modulus (G*) becomes larger and the phase angle becomes smaller. However, when the content of RHB increases from 15% to 20%, the complex shear modulus (G*) decreases and the phase angle increases. These results suggest that the high-temperature rutting resistance of asphalt is improved when the content of RHB is 15%.

**Figure 9:**
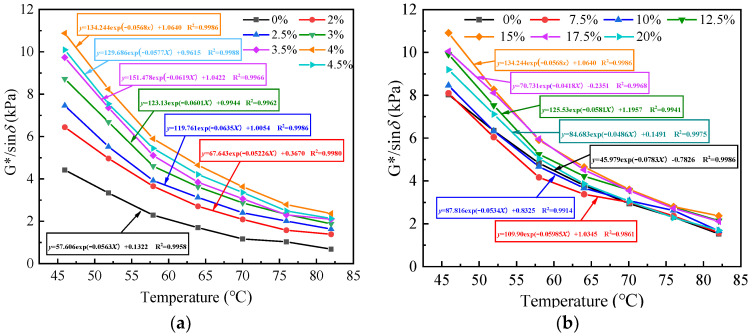
(**a**) Rutting factor of different content of SBS, (**b**) rutting factor of different content of RHB.

The rutting factor (G*/sinδ) characterizes the ability of asphalt to resist permanent deformation. At the same temperature, the larger the G*/sinδ of asphalt, the worse the high-temperature flow deformation of asphalt and the better its rutting resistance. Figure 9a,b shows the curves of the G*/sinδ values versus temperature for the same content of RHB and different content of SBS, and for the same content of SBS and different content of RHB, respectively. As seen from Figure 9a, the overall trend of G*/sinδ gradually increases when the content of SBS increases from 2% to 4%, and G*/sinδ decreases from 4% to 4.5%, indicating that the G*/sinδ is maximized when the content of SBS is 4% and the content of RHB is fixed. Based on this, the influence of RHB content on G*/sinδ was analyzed in this paper when the SBS content was 4%. As can be seen from Figure 9b, the overall trend of G*/sinδ gradually increases when the content of RHB increases from 7.5% to 15%, and G*/sinδ decreases from 15% to 20%, indicating that G*/sinδ reaches its maximum value when the RHB content is 15%. When the content of RHB is 10%, the G*/sinδ value is close to that when the RHB doping is 0%, indicating that the content should not be lower than 10%. It can be seen from Figure 9a,b that the decrease in G*/sinδ varies greatly with the increase or decrease in the RHB or SBS content when the scanning temperature is less than 64 °C. However, when the scanning temperature is higher than 64 °C, the difference of the G*/sinδ decrease is small when the content of RHB or SBS is increased by 2.5%. This means that when the temperature is greater than 64 °C, the influence of the RHB and SBS dosing on the size of G*/sinδ of asphalt is smaller. Overall, the G*/sinδ of composite-modified asphalt is the largest and its anti-rutting performance is the best when the SBS dosing is 4% and the RHB dosing is 15%.

The G*/sinδ curves under different temperatures and variable conditions were fitted, and the fitted curve formulas were all exponential functions in the form of y = A_0_e^R^_0_^x^ + A_1_, in which the exponential coefficient R_0_ had a great influence on G*/sinδ. The exponential coefficient R_0_ ranged from −0.0783 to −0.0418. The larger the value of R_0_, the faster the G*/sinδ curve decreases, and the greater the influence of temperature. In this paper, the optimal fitting curve of the G*/sinδ curve under different content of SBS and RHB is:

G*/sinδ = 134.244e^−0.0577x^ + 1.0640 (1)

The porous structure of RHB make it easier to form a stable skeleton with SBS and matrix asphalt. Additionally, the fiber porous RHB can absorb some of the oil content of the composite-modified asphalt, which can make the composite-modified asphalt harder and increase the elastic ratio. As a result, the rutting resistance of the composite-modified asphalt is improved. The rutting resistance of 15% of RHB is the best, possibly due to the optimal elastic ratio at this level. However, when the RHB content is increased to 20%, the oil content in the composite-modified asphalt decreases significantly, resulting in the asphalt becoming hard and brittle. Consequently, the rutting resistance of the modified asphalt decreases slightly.

#### 3.2.2. BBR

**Figure 10:**
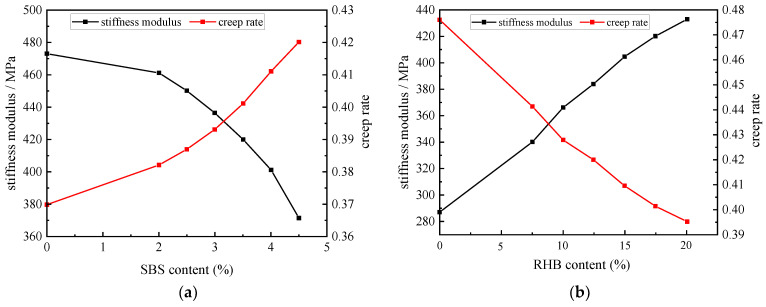
Curves of stiffness modulus and the creep rate of different composite-modified asphalt. (**a**) Composite-modified asphalt with fixed RHB content, (**b**) composite-modified asphalt with fixed RHB content.

The BBR experiment can be used to detect the stiffness modulus and the creep rate of asphalt. The stiffness modulus indicates the deformation ability of asphalt at low temperatures. The greater the stiffness modulus, the worse the deformation ability of asphalt at low temperatures, and the harder and more brittle the asphalt is. The creep rate represents the stress relaxation ability of asphalt at low temperatures. The higher the creep rate, the faster the stress accumulation and dissipation rate of asphalt at low temperatures, and the better the low-temperature performance of asphalt [23]. Figure 10a,b shows the test results obtained by controlling the dosage of RHB and SBS, respectively. The performance of composite-modified asphalt at low temperatures is poor. Increasing the content of RHB would improve the stiffness modulus of asphalt and reduce the creep rate of asphalt, resulting in a decrease in the low-temperature performance of asphalt. Conversely, increasing the content of SBS would improve the low-temperature performance of asphalt. Therefore, the content of RHB in composite-modified asphalt should not exceed 20%.

In summary, a similar analysis of biochar and other biomass carbon material has been reported by other researchers in terms of the high- and low-temperature performance. Previous studies revealed that biochar with a porous fibrous structure can enhance the high-temperature performance of asphalt [24,25], which would slightly impair the low-temperature performance [15].

### 3.3. Microscopic Analysis of RHB-SBS Composite-Modified Asphalt

#### 3.3.1. Fourier Infrared Spectroscopy Test

**Figure 11:**
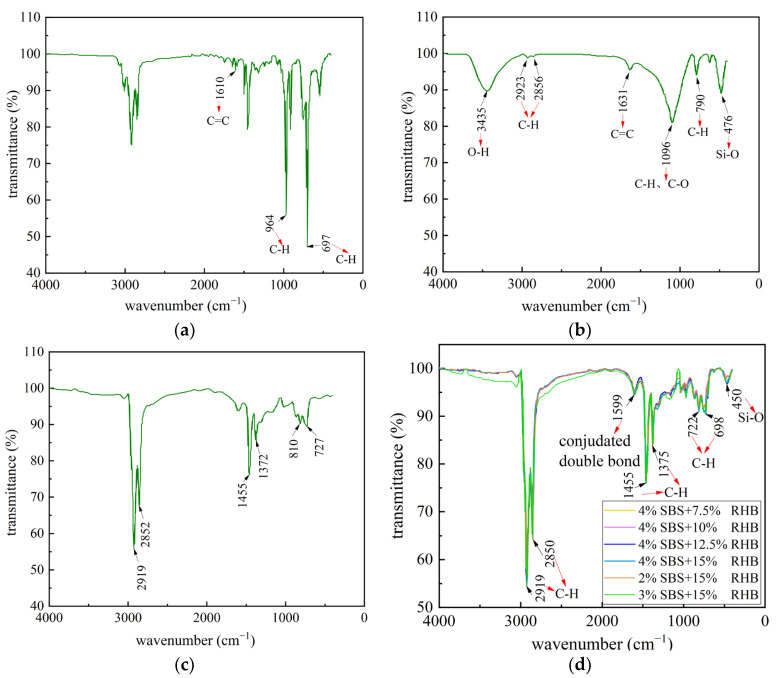
FTIR spectra of modifier and composite-modified asphalt. (**a**) SBS, (**b**) RHB, (**c**) base asphalt, (**d**) comparison of FTIR spectra of composite-modified asphalts with different RHB-SBS contents.

The results of infrared spectrum analysis of RHB, SBS, and composite-modified asphalt with different contents are shown in Figure 11.

The wavenumber range of infrared spectroscopy is 4000~400 cm^−1^, the region of 4000~1400 cm^−1^ is the functional group region, and the main characteristic absorption peak is the intermolecular bending and stretching vibration. The region of 1300~400 cm^−1^ is the fingerprint region, and the main characteristic absorption peak is the vibration between atoms, which is used to distinguish the subtle changes in molecular structure [26]. In Figure 11a, the absorption peaks of SBS are the strongest at 964 cm^−1^ and 697 cm^−1^, which are caused by the wagging of *trans*–C–H and polystyrene block C–H, respectively [27]. The absorption peak at 1610 cm^−1^ resulted from the stretching vibration of the conjugate double bond C=C on the benzene ring skeleton. In Figure 11b, the absorption peak of RHB 3435 cm^−1^ resulted from the stretching vibration of hydroxyl O–H. The absorption peaks at 2923 cm^−1^ and 2856 cm^−1^ are the result of the stretching vibration of C–H on saturated carbon, while the absorption peak at 1631 cm^−1^ is the result of the stretching vibration of olefin C=C. The absorption peak at 1096 cm^−1^ is the result of the in-plane bending vibration of C–H and the stretching vibration of C–O. The absorption peak at 790 cm^−1^ is the result of the out-of-plane bending vibration of double-substituted C–H between benzene rings. Furthermore, the absorption peak at 476 cm^−1^ is the result of the stretching vibration of Si-O. As shown in Figure 11c, the main components of matrix asphalt include cycloalkanes, alkanes, aromatic and heteroatomic derivatives, etc. [28], and the strong absorption peaks mainly exist at 2919 cm^−1^, 2852 cm^−1^, 1455 cm^−1^, 1372 cm^−1^, 810 cm^−1^, and 727 cm^−1^.

In Figure 11d, absorption peaks at 2919 cm^−1^ and 2850 cm^−1^ are the result of the stretching vibration of saturated hydrocarbon C–H, and the absorption peaks at 1599 cm^−1^ are the result of joint absorption vibration of conjugated double bonds. Furthermore, the absorption peaks at 1455 cm^−1^ and 1375 cm^−1^ are the results of the in-plane bending vibration of alkane C–H. The absorption peak at 1031 cm^−1^ is the result of the bending vibration in the C–H plane, and the absorption peak at 965 cm^−1^ is the result of the wagging of *trans*–C–H in SBS. The absorption peaks at 808 cm^−1^, 722 cm^−1^, and 698 cm^−1^ belong to the fingerprint region, where the absorption peaks are caused by the C–H out-of-plane bending vibration, and the absorption peak at 450 cm^−1^ is the result of the Si–O stretching vibration. The absorption peaks at 1031 cm^−1^, 965 cm^−1^, 699 cm^−1^, and 468 cm^−1^ in the composite-modified asphalt appear due to the incorporation of RHB and SBS. These functional groups also exist in RHB and SBS, and no new chemical reactions occur. When the content of SBS increases, the absorption peak generated by Si–O stretching vibration in the fingerprint area shifts from 475 cm^−1^ to 450 cm^−1^, and the absorption peak has a redshift, indicating that the asphalt material structure has changed at this point. The incorporation of RHB causes the absorption peak at this area, and the increase in the SBS content also has an impact on it. This indicates that the crosslinking occurs between SBS and RHB when the asphalt was mixed into them, which changes the overall structure and affects the performance of asphalt. By comparing Figure 11a–d, it was found that the absorption peaks at 3435 cm^−1^ and 1631 cm^−1^ in RHB and 3005 cm^−1^ and 1639 cm^−1^ in SBS disappeared or weakened significantly after composite modification. The results showed that when RHB and SBS modified asphalt, the chemical bond opens in the process of the high-speed shear, and the graft between the asphalt and modifier occurs. However, other researchers have reported that infrared spectroscopy can only qualitatively judge whether the sample contains a certain functional group but cannot explain the number of functional groups [29]. In addition, the thickness of the asphalt samples cannot be identified during the experiment, and the intensity of the absorption peak would be affected by the thickness of the asphalt samples. Therefore, other reasons for the intensity change need to be further studied.

#### 3.3.2. Environmental Scanning Electron Microscopy Test

**Figure 12:**
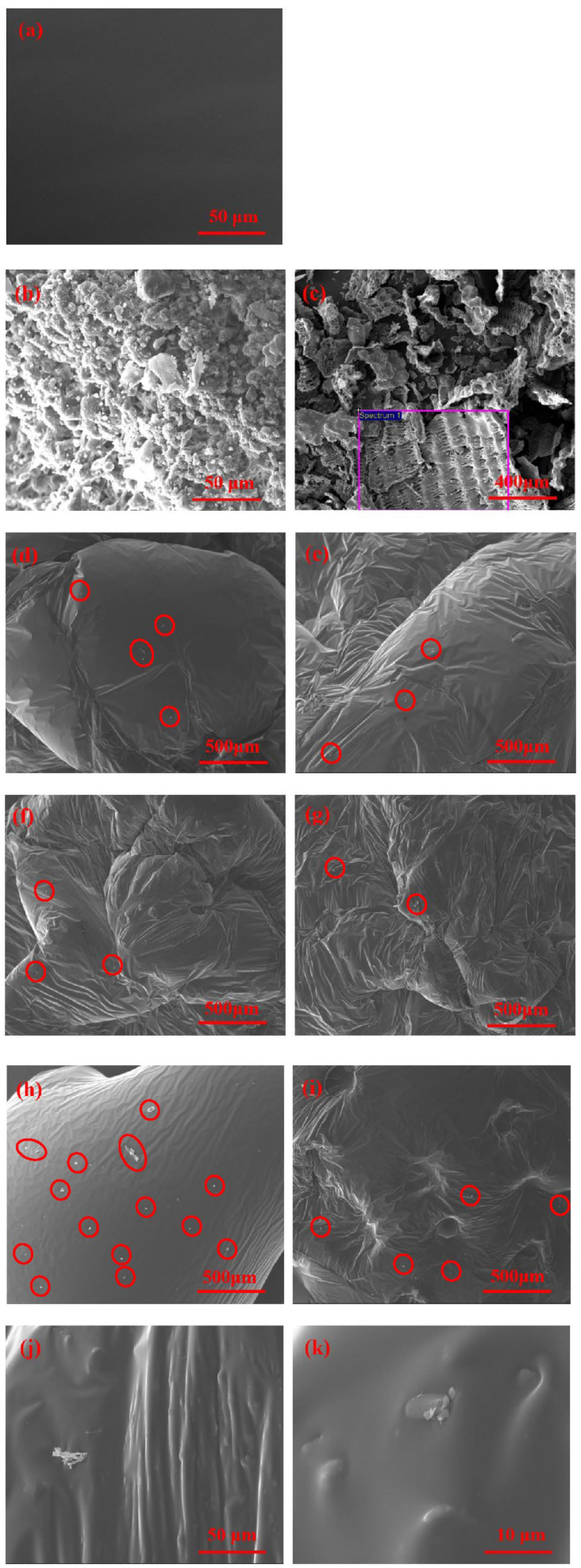
Scanning electron microscopic image of modifier and composite-modified asphalt. (**a**) Base asphalt, (**b**) SBS, (**c**) RHB, (**d**) 7.5% RHB + 4% SBS, (**e**) 10% RHB + 4% SBS, (**f**) 12.5% RHB + 4% SBS, (**g**) 15% RHB + 4% SBS, (**h**) 15% RHB + 2% SBS, (**i**) 15% RHB + 3% SBS, (**j**) Composite-modified asphalt with 800 times magnification, and (**k**) Composite-modified asphalt with 3000 times magnification.

The premise for the modifier to exert its modifying effect on the asphalt is the uniform dispersion in the asphalt, allowing it to transfer its properties to the asphalt [30]. Furthermore, the microscopic morphology and swelling of the modifier in the asphalt can be observed by scanning electron microscopy. The observation results of the microscopic morphology are shown in Figure 12.

In Figure 12a, the matrix asphalt has a uniform and smooth microstructure and homogeneous structure. After the addition of RHB and SBS, the asphalt surface appears to be wrinkling. The SBS particles are completely integrated into the asphalt after high-speed shear, absorbing the light oil in the asphalt and swelling fully to form a new spatial network system. This reduces the movement amplitude of the easiest free components in asphalt under high-temperature conditions, improving the high-temperature stability of the asphalt [30]. After RHB is added to asphalt and sheared at a high speed, its particles are dispersed and most of the particles are coated by asphalt, forming a small bulge with only a few particles that are not fully coated. As a result, the modified asphalt has a heterogeneous structure. After high-speed shear, RHB undergoes desulfurization and degradation, causing the particles to split into smaller sizes and undergo material exchange with the asphalt. Carbon black and silica in RHB enter into the asphalt colloid system, improving the temperature sensitivity of the asphalt [31].

In Figure 12b, the surface of the SBS particles is uneven, with spherical and strip-shaped particles of varying sizes and a large number of internal voids. In Figure 12c, the RHB enlarged 100 times resembles a corn cob in shape [32], with many regular bumps on the outer surface. These bump structures exist in the center of the intersecting grid, and a layer of silica film covers the inner surface. There is a sandwich between the inner and outer surface, which is honeycomb-shaped and contains a large number of pores, and the honeycomb pores are the source of the formation of the activated carbon pores [33]. After mixing RHB with asphalt, the honeycomb pores make RHB have a larger specific surface area and a larger adhesive area between it and the asphalt, which is convenient for the adsorption and wetting of asphalt [34]. The complete wetting of asphalt helps to improve the adhesion force between RHB and asphalt and the viscosity of asphalt.

Figure 12d–g contains scanning electron microscope images at the fixed SBS content of 4% and RHB increased from 7.5% to 15%. The RHB particles that are not completely coated by asphalt can still be observed in Figure 12d, while only a very small amount of RHB particles that are not completely coated by asphalt can be observed in Figure 12e–g. The RHB particles that are not fully coated by asphalt hinder the flow of asphalt and thicken the asphalt. At the same time, the filling effect of RHB particles reduces the low-temperature deformation and the stress relaxation ability of asphalt, leading to a decrease in the low-temperature performance. According to Figure 12g–i, when the RHB content is fixed at 15% and the SBS content increases from 2% to 4%, the RHB particles that are not completely covered by asphalt gradually decrease, and the small bumps increase, indicating that the increase in the SBS content is conducive to the integration of RHB into the asphalt and the improvement of the heterogeneous structure of the composite-modified asphalt. It was found that the modifier in the drum was covered entirely by asphalt after the composite-modified asphalt samples with the same mixture were amplified 800 times and 3000 times. There was adsorption between the modifier and asphalt, and the modifier absorbs part of the oil in the asphalt so that the asphalt component changes, changing the mechanical properties of the asphalt. After RHB and SBS absorb the saturated and aromatic fractions in asphalt, the content of asphaltene and gelatin is relatively increased, and the consistency and viscosity of asphalt are increased. However, there are many bulges and RHB particles that are not completely covered by asphalt, making the asphalt easy to fracture at low temperatures. Therefore, too much RHB in composite-modified asphalt would continuously reduce the low-temperature performance of asphalt.

#### 3.3.3. Leica Fluorescence Electron Microscopy Test

**Figure 13:**
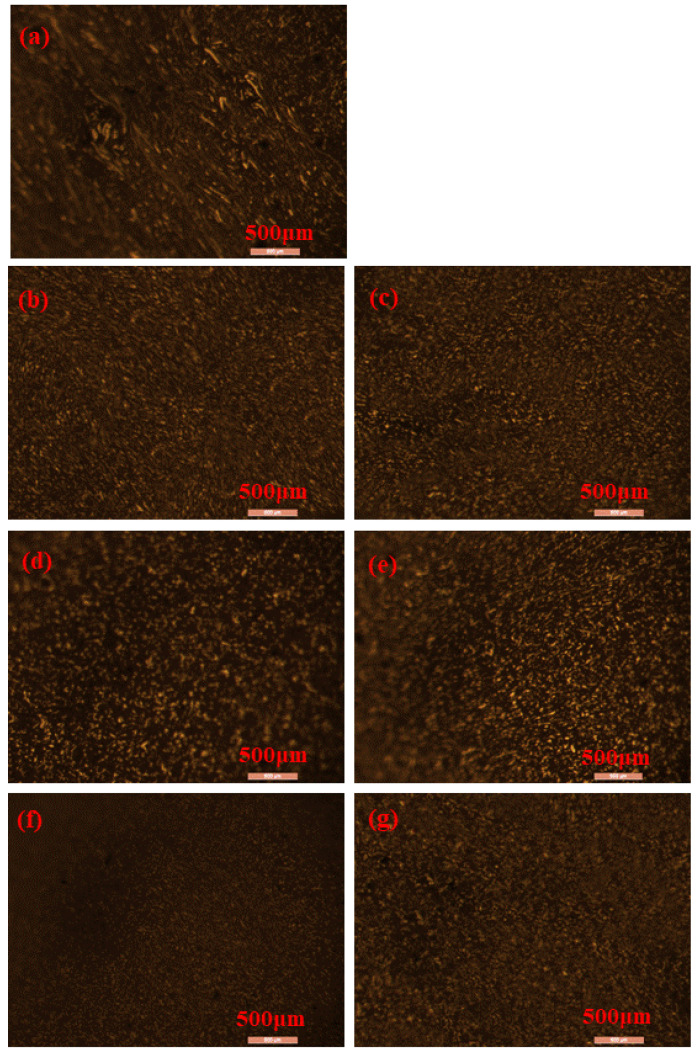
Fluorescence electron microscopy of composite-modified asphalt with different dosages. (**a**) 4% SBS, (**b**) 7.5% RHB + 4% SBS, (**c**) 10% RHB + 4% SBS, (**d**) 12.5% RHB + 4% SBS, (**e**) 15% RHB + 4% SBS, (**f**) 15% RHB + 2% SBS, (**g**) 15% RHB + 3% SBS.

Polymer swelling in modified asphalt forms a polymer phase that emits longer wavelength light when excited by short-wave light waves [35]. When blue light is shone on the modified asphalt, the polymer phase emits yellow light, while the asphalt phase does not emit light. Therefore, fluorescence electron microscopy can be used to observe the actual structure of the polymer phase in asphalt, including the particle size, morphology, distribution characteristics, and the degree of continuity between the asphalt phase and the polymer phase. The phase structure of modified asphalt greatly affects its storage stability, etc., in high-temperature environments [36]. The results of the observations are shown in Figure 13.

Figure 13a shows the fluorescence electron microscopy of a single SBS asphalt. When the content of SBS in asphalt is 4%, the polymer phase after high-speed shear mostly consists of large particle size strip particles, which results in a problem of a large size difference and uneven distribution. Figure 13b–g shows that RHB, SBS, and asphalt become completely miscible after high-speed shear. RHB and SBS absorb light components in asphalt, causing swelling, and a gel mask is generated on the surface, forming a network cross-linked continuous phase system that gives the composite-modified asphalt good storage stability and rheological properties at high temperatures. When the content of SBS is high, there are many large particle size particles in the polymer phase, but the addition of RHB improves the distribution of the large particle size polymer phase, forming an obvious network structure. The light components of asphalt infiltrate into the polymer phase, allowing the asphalt and modifiers to penetrate and adsorb, which is conducive to better modification. When the content of RHB increases, the particle size of the polymer phase in asphalt tends to become larger because the excessive content of RHB increases the adhesion force between the polymer phases, which leads to the inability of RHB to disperse evenly and harms the overall modification effect. After reducing the content of SBS, the polymer phase particles in asphalt become smaller and more evenly distributed. However, the network structure is not obvious compared with the high content of SBS, and the modification effect is relatively poor at this time. Therefore, the content of SBS should not be less than 3%.

### 3.4. Road Performance of Different Types of Asphalt Mixtures

The basic performance indices of the matrix asphalt mixture, single RHB-modified asphalt mixture, and single SBS-modified asphalt mixture are shown in Table 5 [37] and compared with the road performance of the RHB-SBS composite-modified asphalt mixture (the content of RHB is 15% and SBS is 4% with a 15% content and 4% content of SBS).

According to the current specification requirements, the dynamic stability of modified asphalt mixture in hot summer and hot summer areas should be no less than 2400 times/mm. Table 5 shows that the addition of RHB and SBS modifiers to the composite-modified asphalt mixture improves its dynamic stability and high-temperature stability. This is due to the strong cohesion of the composite-modified asphalt, which enhances the temperature sensitivity of the mixture. The RHB particles fill in the asphalt and ore, making the mixture’s skeleton more compact. As a result, the intercalation and extrusion of the composite-modified asphalt mixture are enhanced, making it better able to resist high-temperature rutting deformation. According to the maximum flexural strain in Table 5, compared to the single SBS asphalt mixture, the low-temperature performance of the RHB-SBS composite-modified asphalt mixture decreased, but still meets the specification requirements. Compared with the SBS asphalt mixture, the immersion residual stability ratio and freeze-thaw split intensity ratio of RHB and the SBS composite-modified asphalt mixture are slightly reduced but significantly increased compared with the matrix asphalt mixture and RHB asphalt mixture. This indicates that the incorporation of RHB and SBS can enhance the bonding force between the asphalt and aggregate, preventing the asphalt film from spalling after the action of water, and thus improving the water stability of the asphalt mixture.

## 4. Conclusions

In this study, a matrix asphalt composite modified with different contents of RHB-SBS was characterized using a conventional binder index, rheological behavior, creep behavior, and microstructural characterization tests, and the performance of the mixture was verified. The following primary conclusions were obtained based on the experimental results:

(1) The combination of RHB and SBS increases the softening point and viscosity of composite-modified asphalt, thereby improving the high-temperature deformation resistance of the asphalt binder. When the content of RHB is 15% and SBS is 4%, the phase angle of the composite-modified asphalt is the smallest, the complex shear modulus (G*) and rutting factor are the largest, and the improvement effect of composite-modified asphalt at a high temperature is the most significant.

(2) With the increase in the content of RHB, the stiffness modulus of composite-modified asphalt increases, the creep rate of composite-modified asphalt decreases, and the low-temperature crack resistance of composite-modified asphalt decreases slightly. Therefore, considering the research in this paper, the optimal content of RHB is suggested to be 15%.

(3) The modification of asphalt by RHB and SBS was mainly by physical blending, and a small number of chemical reactions were detected in the modification process. In the infrared spectrogram of the RHB-SBS composite-modified asphalt, the intensity of multiple absorption peaks changed, but no new chemical functional groups appeared. The reason for this is that the chemical bond was opened when the modifier modified the matrix asphalt, and the matrix asphalt and the modifier had a grafting effect.

(4) RHB, with its porous fiber structure, and SBS were swollen when mixed into matrix asphalt, resulting in a gel film and forming a network cross-linked continuous phase system. As a result, asphalt has a good high-temperature storage stability and rheological properties.

It is feasible to reduce the content of SBS and blend in RHB to modify the asphalt. The preparation process of RHB-SBS composite-modified asphalt can be further improved in the future, and systematic research can be conducted on the anti-aging properties and environmental and economic benefits. The impact of both SBS and RHB can also be systematically assessed using design software.

## Figures and Tables

**Table 1:** Basic performance test results of matrix asphalt.

Technical Indicators	Test Results	Technical Requirements	Specification
Penetration (25 °C, 100 g, 5 s) (0.1 mm)	64.3	60~80	T0604-2011
Penetration (30 °C, 100 g, 5 s) (0.1 mm)	106.1	--	T0604-2011
Penetration (15 °C, 100 g, 5 s) (0.1 mm)	25.3	--	T0604-2011
Softening point (°C)	47.2	≥43	T0606-2011
Ductility (5 cm/min, 10 °C) (cm)	39.8	≥20	T0605-2011
Dynamic viscosity (60 °C) (Pa·s)	218.3	≥160	T0620-2000
Density (15 °C) (g/cm^3^)	0.97	--	T0603-2011

**Table 2:** Basic physical properties of SBS.

Sample	S/B	Tensile Strength (MPa)	Stress at Definite Elongation (MPa)	Elongation (%)	Permanent Deformation (%)
YH-791-H SBS	30/70	≥18	≥1.9	≥700	≤45

**Table 3:** Component analysis of rice husk biochar.

Sample	Elemental Analysis/%					Component Analysis/%			
	C	H	O	N	S	Moisture	Ash Content	Volatile Component	Fixed Carbon
RHB	51.68	1.77	45.72	0.64	0.19	5.60	38.13	9.65	53.34

**Table 4:** Basic technical indicators of rice husk biochar.

Sample	pH	Water Content (%)	Carbon Content (%)	Specific Surface Area (m^2^/g)	Bulk Specific Weight (mg/mL)
RHB	>7	<10	>50	>400	<450

**Table 5:** The road performance of different asphalt mixtures.

Type of mixture	Dynamic Stability/Time·mm^−1^	Maximum Flexural Strain/με	Immersion Residual Stability Ratio/%	Freeze-Thaw Split Intensity Ratio/%
Base asphalt mixture	1796	2159	61.2	66.8
RHB asphalt mixture	3691	1068	63.5	68.7
SBS asphalt mixture	6592	3805	86.3	87.8
RHB-SBS composite-modified asphalt mixture	7715	3224	82.9	87.2

## Data Availability

Data will be made available on request.
